# AI in Hand and Wrist Radiography: Multimodal Large Language Models for Distal Radius Fracture Detection and Characterization

**DOI:** 10.3390/diagnostics16081171

**Published:** 2026-04-15

**Authors:** Ibrahim Güler, Armin Kraus, Gerrit Grieb, David Breidung, Martin Lautenbach, Henrik Stelling

**Affiliations:** 1 Department of Plastic, Aesthetic and Hand Surgery, Otto-von-Guericke University, 39120 Magdeburg, Germany; armin.kraus@med.ovgu.de; 2 Department of Health Management, Friedrich-Alexander-Universität Erlangen-Nürnberg (FAU), Lange Gasse 20, 90403 Nürnberg, Germany; henrikstelling@googlemail.com; 3 Department of Plastic Surgery and Hand Surgery, Gemeinschaftskrankenhaus Havelhoehe, Kladower Damm 221, 14089 Berlin, Germany; gerritgrieb@gmx.de; 4 Department of Plastic Surgery and Hand Surgery, Medical Faculty, RWTH Aachen University, Pauwelsstrasse 30, 52074 Aachen, Germany; 5 Department of Health, University of Witten/Herdecke, 58455 Witten, Germany; david.breidung@usz.ch; 6 Department of Plastic Surgery and Hand Surgery, University Hospital Zurich, Rämistrasse 100, 8091 Zurich, Switzerland; 7 Department of Hand Surgery, Orthopedics, and Trauma Surgery, Waldfriede Hospital, Argentinische Allee 40, 14163 Berlin, Germany; m.lautenbach@waldfriede.de; 8 Practices for Nuclear Medicine, Rubensstraße 125, 12157 Berlin, Germany

**Keywords:** multimodal large language models, generative artificial intelligence, orthopedic radiography, distal radius fracture, fracture detection, intra-articular extension, fracture displacement, sensitivity, inter-run reliability, medical image interpretation

## Abstract

**Background/Objectives**: Multimodal large language models (MLLMs) are increasingly evaluated for diagnostic tasks in medical imaging, including radiographic interpretation. However, most studies focus primarily on binary fracture detection and rarely assess clinically relevant fracture characteristics such as displacement or intra-articular extension, which influence treatment decisions. In addition, most evaluations rely on single-run inference designs that do not assess response reproducibility. This study evaluated the diagnostic performance and inter-run reliability of five MLLMs for radiographic assessment of distal radius fractures. **Methods**: Fifty fracture-positive distal radius radiographs were evaluated by five MLLMs (ChatGPT 5.3, Gemini 3.1 Pro, Claude Opus 4.6, Grok 4.1, and ERNIE 5.0) across five independent zero-shot inference runs (*n* = 1250 observations). Diagnostic tasks included fracture detection, intra-articular extension, and displacement. Sex and age were exploratory endpoints. Performance was summarized using sensitivity (fracture detection) and accuracy (other tasks), with inter-run reliability assessed via Fleiss’ κ. **Results**: Performance varied across tasks and models. Fracture detection sensitivity ranged from 39.6% to 99.6%, with two models exceeding 90%. Intra-articular extension accuracy ranged from 51.6% to 55.6%, consistent with chance-level performance. Displacement classification ranged from 34.8% to 70.4%. One model achieved substantial inter-run agreement across binary tasks (κ > 0.60), whereas two models showed slight agreement (κ < 0.20). **Conclusions**: Only two models exceeded 90% sensitivity for fracture detection, while intra-articular extension remained at chance level (≤55.6%). Substantial inter-run reliability (κ > 0.60) was observed in only one model. These findings indicate that current MLLMs do not reliably support multidimensional fracture assessment and that single-run evaluations overestimate robustness.

## 1. Introduction

Distal radius fractures are among the most common fractures encountered in emergency and orthopedic settings, accounting for approximately one-sixth of all fractures treated in emergency departments [1,2,3]. Beyond the binary question of fracture presence, clinical management critically depends on the accurate characterization of fracture morphology: whether the fracture extends into the radiocarpal joint (intra-articular involvement) and whether clinically relevant displacement is present. These features directly influence the choice between conservative and operative treatment and carry prognostic implications for functional recovery [4,5,6,7].

Multimodal large language models (MLLMs), which extend large language models (LLMs) by integrating both textual and visual inputs and outputs, have recently attracted attention as potential artificial intelligence (AI)-based decision-support tools in radiology. Unlike task-specific convolutional neural networks (CNNs), such as fracture-detection systems like BoneView^®^ (Gleamer, Paris, France), which are optimized for narrowly defined classification tasks, MLLMs operate as general-purpose reasoning systems capable of interpreting medical images while producing natural language outputs. A growing body of literature has examined the potential of AI-based systems in musculoskeletal diagnostics, including ligament injury detection, osteoarthritis grading, bone age assessment and fracture classification, reporting heterogeneous results that range from near-expert accuracy to performance below chance level [8,9,10,11,12,13,14,15,16,17].

However, two fundamental limitations pervade the current evidence base. First, most evaluations rely on single-run inference, implicitly treating generative models as deterministic systems. This assumption is unjustified: identical inputs may yield divergent outputs across repeated inferences due to stochastic decoding processes. Although the underlying model architectures are deterministic, real-world deployments rely on sampling-based decoding strategies that intentionally introduce stochasticity into individual responses. As a result, repeated queries may produce variable outputs despite identical inputs, reflecting the operational characteristics of MLLMs rather than random system instability. Yet the resulting inter-run reliability remains largely unquantified. Recent work on MLLM repeatability in diagnostic reasoning has confirmed that accuracy and consistency represent distinct performance dimensions, with reproducibility not correlating with correctness across repeated inferences. Notably, models may exhibit high reproducibility despite low accuracy, reflecting systematic error, or intermittent correctness without consistency, indicating probabilistic instability [18,19].

Second, existing studies often reduce fracture assessment to a binary detection task (fracture vs. no fracture). In clinical practice, however, the diagnostic challenge extends well beyond determining whether a fracture is present. Characterizing fracture features (joint involvement, displacement, fragment configuration) requires a qualitatively different level of radiographic interpretation, involving spatial reasoning across multiple projections and integration of subtle cortical and trabecular signs. Whether MLLMs can perform this graded diagnostic reasoning, and whether they do so with any degree of consistency, has not been systematically examined [20,21,22,23].

The present study addresses both gaps by extending the multi-run evaluation framework to a multi-task setting. Five frontier MLLMs were assessed on 50 distal radius fracture cases across five independent inference runs each, yielding 1250 total inferences. Models were evaluated not only for fracture detection but also for intra-articular extension, fracture displacement, and as exploratory secondary endpoints, demographic attribute inference (patient age and sex). By jointly analyzing accuracy and inter-run reliability across tasks of increasing diagnostic complexity, this study provides a structured assessment of whether current MLLMs can move beyond binary fracture detection toward clinically meaningful fracture characterization.

## 2. Materials and Methods

### 2.1. Study Design and Objectives

This experimental benchmarking study evaluated the diagnostic performance of five MLLMs in the radiographic assessment of distal radius fractures using a repeated-run inference framework. No patient-level clinical data were collected, and no clinical validation was performed. The study design and reporting structure followed the Checklist for Artificial Intelligence in Medical Imaging (CLAIM) guidelines [24], as applicable to the evaluation of pre-trained foundation models. The study was designed to characterize diagnostic performance, inter-run reliability (i.e., agreement across repeated inference runs of the same model), and response stability across multiple independent inference runs rather than to establish clinical validation or population-level diagnostic performance.

The study had three primary objectives: (1) to quantify diagnostic performance across five independent inference runs per case for five distinct diagnostic tasks; (2) to assess inter-run reliability across repeated runs using Fleiss’ kappa (κ); and (3) to evaluate the relationship between diagnostic accuracy and response consistency across models and tasks [25].

### 2.2. Dataset and Case Selection

First, 50 fracture-positive distal radius radiograph cases were selected from publicly available, curated educational radiology resources (https://www.radiopaedia.org, accessed on 2 March 2026) [26]. Cases were accessed and analyzed in compliance with the platform’s Terms of Use and are licensed under the Creative Commons Attribution-NonCommercial-ShareAlike 3.0 Unported License (CC BY-NC-SA 3.0). Case selection was performed manually by the authors according to predefined qualitative criteria. No images were reproduced or redistributed as part of this study.

Inclusion criteria were: (1) availability of standardized radiographic projections comprising at least two orthogonal views (posteroanterior and lateral) of the distal radius; in many cases an additional oblique projection was available and included when present; (2) absence of superimposed annotations (e.g., arrows, circles, or text overlays), with the exception of standard anatomical side markers (L/R); (3) diagnostic-quality images in web-optimized formats (JPEG/PNG) with sufficient native resolution to allow visual assessment of cortical margins, trabecular architecture, and fracture lines; (4) availability of basic demographic metadata including patient age and sex; and (5) availability of an unambiguous reference diagnosis including intra-articular extension and displacement status, as stated in the original case description (i.e., cases with inconclusive or discussion-based diagnoses were excluded). As cases originated from multiple contributing institutions, device variability across radiographs was inherent to the dataset and not controlled. To mitigate potential effects on diagnostic reference quality, cases were pre-filtered using the source database’s built-in case categorization [26] and subsequently reviewed individually by all authors, comprising three senior hand surgeons, two hand surgery residents, and one nuclear medicine resident, who reached consensus on each diagnostic classification.

The final cohort comprised 50 confirmed distal radius fractures with a sex distribution of 56.0% male and 44.0% female. All cases were fracture-positive by design, enabling focused analysis of model behavior under conditions of confirmed pathology. This design intentionally focuses on characterization tasks (intra-articular extension and displacement) that require fracture-positive cases. Specificity and negative predictive value (NPV) were therefore not assessed.

### 2.3. Models Under Evaluation

Five frontier multimodal large language models (MLLMs), a subclass of generative artificial intelligence (GenAI) systems, were evaluated to represent a broad spectrum of proprietary and international AI platforms:

- Claude Opus 4.6 (Anthropic, San Francisco, CA, USA; proprietary)
- ChatGPT 5.3 (OpenAI, San Francisco, CA, USA; proprietary)
- Gemini 3.1 Pro (Google DeepMind, Mountain View, CA, USA; proprietary)
- Grok 4.1 (xAI, San Francisco, CA, USA; proprietary)
- ERNIE 5.0 (Baidu, Beijing, China; proprietary)

All models were accessed via their official web interfaces using default inference settings. No Application programming interface (API)-level parameter modifications (e.g., temperature, top-p, or decoding strategies) were applied. This approach was chosen to approximate real-world usage conditions and to reflect how these systems are typically accessed by clinicians and researchers in practical settings. Model versions reflect the publicly available system state at the time of data collection in March 2026.

### 2.4. Prompting Strategy and Inference Protocol

Each model received five inference runs per case using an identical, standardized zero-shot prompt. The full prompt text is provided in Supplementary Material S1. The prompt instructed the model to analyze the provided radiograph and report: (1) binary fracture detection (yes/no); (2) intra-articular extension (yes/no); (3) fracture displacement (yes/no); (4) estimated patient age; and (5) inferred patient sex (male/female). The standardized prompt required structured categorical responses in a predefined output format (yes/no for binary tasks; male/female for sex classification; numeric value for age). All model outputs across all inference runs conformed to this format, yielding unambiguous categorical assignments without the need for post hoc interpretation or reclassification of free-text responses.

To ensure independence between runs, each inference was conducted in a fresh session without prior conversational context. No conversational memory, feedback, or adaptive prompting was permitted. Each run was treated as an independent realization of the model’s diagnostic response under identical input conditions.

### 2.5. Outcome Definitions

Primary outcomes included: (1) diagnostic performance for each of the four binary classification tasks, reported as mean sensitivity for fracture detection (given the fracture-positive-only design) and mean classification accuracy for intra-articular extension, fracture displacement, and sex classification (where both positive and negative ground truth labels were represented); and (2) inter-run reliability for each binary task, quantified using Fleiss’ kappa (κ) to assess agreement across repeated runs. Displacement classification followed a two-stage process. First, cases were pre-filtered using the source database [26]. Subsequently, all authors independently reviewed each case and reached consensus on displacement status based on the presence of radial shortening, dorsal tilt, and/or intra-articular step-off. This approach reflects established radiographic criteria for displacement assessment in distal radius fractures [5,7], although no single quantitative threshold (e.g., >2 mm articular step-off) was applied as a rigid cutoff, as such thresholds pertain to surgical indication rather than to the binary classification of displacement.

Secondary outcomes included: (1) age estimation accuracy, quantified using the Mean Absolute Error (MAE) between predicted and ground truth age values calculated across all predictions (*n* = 250 per model); and (2) task-specific accuracy–consistency relationships across models and tasks.

### 2.6. Statistical Analysis

All statistical analyses were performed using Python 3.12 with standard statistical and data visualization libraries. Diagnostic performance for each binary task was summarized descriptively as the mean accuracy across five inference runs, with the corresponding range (minimum–maximum) reported to illustrate inter-run variability. Inter-run variability (i.e., the spread of aggregate accuracy across repeated runs) and inter-run reliability (i.e., case-level agreement across runs, quantified by Fleiss’ κ) were analyzed as complementary but distinct performance dimensions.

Inter-run reliability was assessed using Fleiss’ kappa (κ). Repeated inference runs of the same model do not constitute independent raters in the classical psychometric sense; Fleiss’ κ is therefore used here as a descriptive measure of output consistency across repeated stochastic inferences rather than as a measure of inter-observer agreement between independent human raters. κ values were interpreted according to the conventional Landis–Koch thresholds [27]: <0.20 slight, 0.21–0.40 fair, 0.41–0.60 moderate, 0.61–0.80 substantial, and 0.81–1.00 almost perfect agreement. Fleiss’ κ is sensitive to prevalence effects. When the marginal distribution of ratings is highly imbalanced, for example when a model achieves near-perfect agreement in a fracture-positive-only dataset, the expected chance agreement (P_e_) approaches 1, which can mathematically depress κ despite high observed agreement. This phenomenon, described by Feinstein and Cicchetti as the “kappa paradox”, was considered when interpreting reliability estimates [28,29].

Age estimation accuracy was quantified using the mean absolute error (MAE ± SD) between model-predicted and ground truth ages across all five runs (*n* = 250 observations per model). For sex classification, a majority-class baseline was calculated: given the cohort sex distribution (56.0% male), a naive classifier predicting “male” for every case would achieve 56.0% accuracy, serving as a reference threshold for evaluating model performance. For intra-articular extension and displacement, the majority-class baselines were both 62.0% (31/50 cases in the majority class for each task). References to “chance level” in Section 3 denote the 50% threshold expected under random binary classification and should be interpreted in the context of these class distributions. To quantify the precision of diagnostic performance estimates, 95% confidence intervals were calculated using the Wilson score method for pooled observations across all five runs (*n* = 250 per model per task). Confidence intervals should be interpreted descriptively, as pooling across repeated evaluations of identical cases does not fully satisfy strict independence assumptions.

Given the exploratory hypothesis-generating nature of this benchmarking study and the repeated-measures design, classical inferential testing and specificity-based metrics were not performed. ROC analysis was not applicable due to the absence of true negative cases for fracture detection and the lack of probability-scored outputs for other binary tasks. Inferential model comparisons (e.g., McNemar test) were not conducted, as repeated stochastic runs on identical cases violate the independence assumption required for such tests.

## 3. Results

### 3.1. Fracture Detection

**Figure 1:**
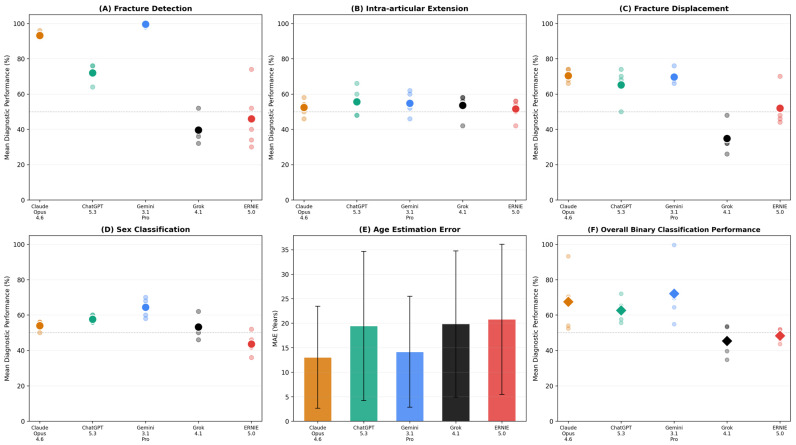
Multi-panel overview of model performance across diagnostic tasks. Each small translucent dot represents diagnostic performance from one of five independent inference runs per model; large colored dots indicate mean performance across runs. (**A**): fracture detection (sensitivity); (**B**): intra-articular extension; (**C**): fracture displacement; (**D**): sex classification; (**E**): age estimation error (bars = MAE, whiskers = SD); (**F**): overall binary classification performance (diamonds = mean across four binary tasks, small dots = individual task metrics). The horizontal dashed line in panels A–D and F marks 50% as a visual reference. Model colors: Claude Opus 4.6 (orange), ChatGPT 5.3 (green), Gemini 3.1 Pro (blue), Grok 4.1 (black), ERNIE 5.0 (red). MAE = Mean Absolute Error; SD = Standard Deviation.

Sensitivity for binary fracture detection varied substantially across models (Table 1, Figure 1A). Gemini 3.1 Pro achieved near-perfect sensitivity (99.6%; range: 98.0–100.0%), followed by Claude Opus 4.6 (93.2%; range: 92.0–96.0%). ChatGPT 5.3 demonstrated intermediate performance (72.0%; range: 64.0–76.0%), while ERNIE 5.0 (46.0%; range: 30.0–74.0%) and Grok 4.1 (39.6%; range: 32.0–52.0%) performed near chance level.

ERNIE 5.0 exhibited the largest run-to-run variability in this task (44 percentage-point spread), suggesting pronounced probabilistic instability (i.e., large variability across repeated inference runs). In contrast, both Gemini 3.1 Pro and Claude Opus 4.6 maintained narrow performance bands. This indicates that fracture detection performance is relatively stable across runs for high-performing models, whereas lower-performing models show substantially greater run-to-run variability.

### 3.2. Intra-Articular Extension

Classification of intra-articular extension proved substantially more challenging (Table 2, Figure 1B). All five models performed near chance level, with mean accuracies ranging from 51.6% (ERNIE 5.0) to 55.6% (ChatGPT 5.3). No model consistently exceeded chance-level performance across all runs. Claude Opus 4.6 (52.4%; range: 46.0–58.0%), Gemini 3.1 Pro (54.8%; range: 46.0–62.0%), and Grok 4.1 (53.6%; range: 42.0–58.0%) exhibited comparable intermediate performance. Classification performance remained near chance level (~50%) but consistently below the majority-class baseline of 62.0%, indicating that models did not exceed naive prediction.

### 3.3. Fracture Displacement

Displacement assessment showed a wider performance spread across models (Table 3, Figure 1C). Claude Opus 4.6 achieved the highest accuracy (70.4%; range: 66.0–74.0%), closely followed by Gemini 3.1 Pro (69.6%; range: 66.0–76.0%) and ChatGPT 5.3 (65.2%; range: 50.0–74.0%). ERNIE 5.0 performed near chance level (52.0%; range: 44.0–70.0%), and Grok 4.1 fell below it (34.8%; range: 26.0–48.0%). Notably, ChatGPT 5.3 and ERNIE 5.0 exhibited substantial run-to-run variability (24 and 26 percentage-point spreads, respectively), indicating inconsistent displacement assessment behavior. Performance on this task varied more widely across models than for other subtasks, with higher-performing models achieving moderate accuracy while others remained near or below chance level. Relative to the majority-class baseline of 62.0%, three models (Claude Opus 4.6, Gemini 3.1 Pro, ChatGPT 5.3) exceeded naive classification, while ERNIE 5.0 and Grok 4.1 remained below it.

### 3.4. Accuracy–Consistency Dissociation

**Figure 2:**
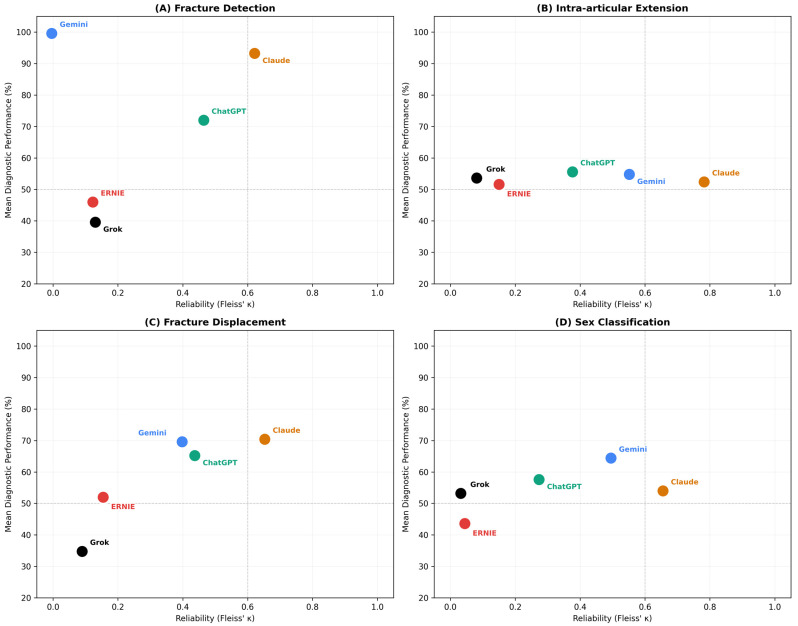
Task-specific accuracy–consistency profiles. Models are plotted according to mean diagnostic performance (sensitivity for fracture detection, classification accuracy for other tasks; *y*-axis) and inter-run reliability measured by Fleiss’ κ (*x*-axis). Dashed lines indicate proposed thresholds: 50% accuracy (horizontal) and κ = 0.60 corresponding to substantial agreement (vertical). Panel (**A**): fracture detection; (**B**): intra-articular extension; (**C**): fracture displacement; (**D**): sex classification. Note: For fracture detection (Panel A), Gemini 3.1 Pro achieved near-perfect sensitivity (99.6%), resulting in a near-zero κ due to the kappa paradox (see Section 2.6). κ = Fleiss’ kappa.

Fleiss’ κ analysis revealed marked differences in inter-run reliability across tasks and models (Table 4, Figure 2). The relationship between diagnostic accuracy and inter-run reliability demonstrated task-dependent accuracy–consistency dissociation patterns (i.e., divergence between diagnostic accuracy and inter-run reliability).

Claude Opus 4.6 was the only model to achieve substantial inter-run reliability across all evaluated tasks (κ range: 0.621–0.782). For fracture detection, Claude Opus 4.6 demonstrated substantial agreement (κ = 0.621), while ChatGPT 5.3 reached moderate levels (κ = 0.464). Grok 4.1 and ERNIE 5.0 exhibited only slight agreement (κ = 0.130 and 0.122, respectively), reflecting unstable run-to-run behavior. Gemini 3.1 Pro represents a special case: despite achieving the highest sensitivity (99.6%), its κ value approached zero due to the kappa paradox inherent to near-ceiling performance in a fracture-positive-only dataset (see Section 2.6 and Section 4.2). Inspection of individual run-level outputs confirmed that Gemini 3.1 Pro misclassified a single case in one of five runs, while all remaining cases were consistently classified across all runs.

For intra-articular extension, Claude Opus 4.6 again achieved the highest reliability (κ = 0.782, substantial agreement) despite only moderate accuracy (52.4%), indicating reproducible but frequently incorrect classifications, a pattern consistent with systematic error. Gemini 3.1 Pro (κ = 0.551, moderate) and ChatGPT 5.3 (κ = 0.376, fair) demonstrated intermediate consistency. Grok 4.1 (κ = 0.081) and ERNIE 5.0 (κ = 0.150) showed only slight agreement, reflecting near-random inter-run behavior.

For displacement assessment, Claude Opus 4.6 achieved the most favorable accuracy–consistency profile (accuracy: 70.4%, κ = 0.653, substantial agreement). ChatGPT 5.3 (κ = 0.437, moderate) and Gemini 3.1 Pro (κ = 0.398, fair) demonstrated intermediate reliability alongside comparable accuracy levels (65.2% and 69.6%, respectively). Notably, Gemini 3.1 Pro combined relatively high accuracy with only fair consistency, suggesting that correct displacement classifications were not reliably reproduced across runs. Grok 4.1 (κ = 0.089) and ERNIE 5.0 (κ = 0.154) exhibited slight agreement with low accuracy.

For sex classification, Claude Opus 4.6 achieved substantial reliability (κ = 0.655) but only marginally below the majority-class baseline accuracy (54.0% vs. 56.0%). Gemini 3.1 Pro was the only model to meaningfully exceed the majority-class baseline (accuracy: 64.4%, κ = 0.495, moderate). ChatGPT 5.3 showed fair agreement (κ = 0.273) with near-baseline accuracy (57.6%). Grok 4.1 (κ = 0.032) and ERNIE 5.0 (κ = 0.044) approximated random guessing in both accuracy and consistency.

### 3.5. Exploratory Demographic Attribute Inference

As a secondary analysis, model outputs were examined for implicit inference of patient age and sex across all five runs (*n* = 250 observations per model). Performance was generally poor (Table 5).

For age estimation, Claude Opus 4.6 achieved the lowest MAE (13.04 ± 10.40 years), followed by Gemini 3.1 Pro (14.16 ± 11.32 years). ChatGPT 5.3 (19.43 ± 15.22 years), Grok 4.1 (19.84 ± 14.91 years), and ERNIE 5.0 (20.79 ± 15.31 years) exhibited larger deviations. Age estimation errors exceeded one decade across all evaluated models.

Sex prediction accuracy ranged from 43.6% (ERNIE 5.0) to 64.4% (Gemini 3.1 Pro). Given the male predominance in the study cohort (56.0%), a majority-class classifier predicting “male” for every case would achieve 56.0% accuracy. Only Gemini 3.1 Pro (64.4%) and ChatGPT 5.3 (57.6%) exceeded this baseline, while Claude Opus 4.6 (54.0%), Grok 4.1 (53.2%), and ERNIE 5.0 (43.6%) performed at or below the level of naive classification.

## 4. Discussion

This study evaluated the diagnostic performance and inter-run reliability of five contemporary MLLMs on radiographic assessment of distal radius fractures across five independent inference runs, yielding 1250 total observations. The results demonstrate a clear task-dependent performance hierarchy, with fracture detection emerging as the most tractable task for current MLLMs, while clinically critical subtasks such as intra-articular extension assessment remain at chance level. Critically, the multi-run design uncovered substantial dissociations between accuracy and inter-run reliability that would have been entirely obscured by conventional single-run evaluation.

### 4.1. Task-Dependent Performance Hierarchy

Fracture detection emerged as the task most amenable to current MLLM capabilities. Two of the five models achieved sensitivities above 90%, consistent with prior evaluations of MLLMs on fracture identification tasks where leading models have demonstrated high sensitivity for visually unambiguous pathology [8,9,10,11,12,13,14,20,21,22,23]. The strong performance on fracture detection likely reflects the visual salience of cortical disruption in distal radius fractures, which presents a relatively unambiguous binary signal compared with the subtler findings required for subtask classification.

In contrast, intra-articular extension proved uniformly difficult, with all five models performing at or near chance level (~50%) and consistently below the majority-class baseline for this task (62.0%). Identifying articular involvement on standard radiographs requires integration of information across multiple projections and detection of subtle cortical continuity changes at the articular surface, a level of spatial reasoning that current vision encoder architectures appear unable to perform reliably. The clinical significance of this limitation is substantial, as intra-articular involvement is a key determinant of operative management and long-term prognosis in distal radius fractures [4,5,6,7]. The uniform failure across different models suggests a shared architectural limitation rather than a model-specific deficiency, indicating that current vision encoders do not yet support the resolution and cross-projection integration required for this task.

Displacement classification showed intermediate and more differentiated performance. Leading models demonstrated meaningful diagnostic capability above chance, yet fell short of the reliability required for unsupervised clinical deployment, while lower-performing models remained near or below chance level. Unlike intra-articular extension, displacement features such as angulation and shortening may produce more visually salient radiographic changes, which likely explains the comparatively higher, though still insufficient, performance on this task. This gradient suggests that displacement assessment occupies a transitional zone between tasks that current MLLMs can and cannot reliably perform.

### 4.2. Accuracy–Consistency Dissociation

A central finding of this study is the dissociation between diagnostic accuracy and inter-run reliability, a pattern previously described in MLLM evaluation contexts [18,19]. Only one model achieved consistently substantial inter-run agreement across all binary tasks (κ > 0.60), indicating reproducible case-level responses across repeated evaluations. By contrast, the highest-performing model on fracture detection exhibited near-zero κ on this task. This finding reflects a statistical artefact rather than true instability: in a fracture-positive-only dataset where the model identifies nearly all cases correctly, the marginal distribution of ratings becomes strongly imbalanced, causing expected chance agreement (P_e_) to approach 1 and mathematically depressing κ regardless of actual consistency. This phenomenon, described by Feinstein and Cicchetti as the kappa paradox [28,29], should be interpreted as a prevalence-driven ceiling effect on κ rather than evidence of unreliable model behavior on this specific task. On other tasks where prevalence imbalance was less extreme, this model demonstrated only moderate agreement, indicating less stable case-level responses than its aggregate accuracy would suggest.

Two models showed consistently slight agreement across all tasks (κ < 0.20), indicating that their outputs were largely non-reproducible across runs. For these models, any individual inference result carries minimal predictive value for a subsequent query on the same case. This pattern of probabilistic instability, defined as the tendency to produce divergent outputs for identical inputs across independent sessions, represents a fundamental barrier to clinical utility that aggregate accuracy metrics fail to capture.

These findings reinforce the argument that multi-run evaluation protocols are essential for characterizing MLLM behavior in diagnostic contexts. Single-run assessments would have identified the top-performing model on fracture detection without revealing its less consistent case-level behavior on other tasks and would have obscured the systematic unreliability of lower-ranked models [18,19,30].

More broadly, these results challenge the assumption that single-run accuracy adequately characterizes model performance. The present results demonstrate that reproducibility constitutes an independent dimension of diagnostic evaluation that is not captured by aggregate accuracy metrics. A model that is accurate on average but not reproducible across individual inferences cannot be considered reliable in a clinical context, where each patient encounter requires a dependable assessment. Integrating reliability assessment into MLLM evaluation frameworks is therefore essential for any meaningful translation toward clinical application.

### 4.3. Demographic Attribute Inference

Sex classification and age estimation were included as exploratory tasks to probe the extent to which MLLMs extract demographic information from skeletal radiographs. No model meaningfully exceeded the majority-class baseline for sex classification, and age estimation errors were substantial across all models. These tasks were not intended as clinically motivated assessments but rather as probes of visual feature extraction depth. The results confirm that current architectures do not encode sufficient bone morphology or density information to support demographic inference from plain radiographs.

### 4.4. Comparison with Prior Literature

The present findings extend the growing body of literature evaluating AI performance on musculoskeletal radiograph interpretation. While prior studies have predominantly evaluated single models on fracture detection using single-run designs, this study contributes a multi-model, multi-run framework that jointly assesses accuracy and reproducibility across multiple diagnostic dimensions. The task-dependent performance hierarchy observed here is broadly consistent with reports that MLLMs perform best on visually salient binary classification tasks and struggle with tasks requiring spatial reasoning or nuanced anatomical interpretation [8,9,10,11,12,13,14,20,21,22,23].

Dedicated CNN-based fracture detection systems such as BoneView^®^ have demonstrated high diagnostic performance on distal radius radiographs, with reported accuracies exceeding 90% in controlled datasets [31]. However, such systems are trained for narrowly defined classification tasks and do not provide the flexible, multimodal reasoning capabilities characteristic of foundation-model-based MLLMs. The present evaluation therefore addresses a complementary question: not whether AI can detect fractures per se, but whether general-purpose MLLMs can perform the multidimensional radiographic assessment required in clinical fracture management.

More broadly, CNN-based and transformer-based architectures represent complementary paradigms in medical image analysis [32]. CNNs are data-efficient and well suited for narrowly defined tasks such as single-region fracture detection, but their fixed receptive fields limit generalization across tasks. Transformer-based architectures, which underlie current MLLMs, require substantially larger training datasets but can capture global context across an image and transfer learned representations more flexibly to new tasks. Which paradigm will ultimately prevail in diagnostic imaging, or whether hybrid approaches combining both will dominate, remains an open question.

### 4.5. Clinical Implications

From a clinical perspective, the results support a cautious interpretation of MLLM capabilities in radiographic fracture assessment. While fracture detection by leading models may approach levels that could supplement clinical workflows under constrained conditions, the inconsistent performance on subtask classification and the substantial inter-run variability observed in most models preclude unsupervised deployment. In particular, the uniform failure on intra-articular extension classification highlights a critical gap between current MLLM capabilities and the multidimensional assessments required for fracture management decisions [4,5,6,7]. In surgical management of distal radius fractures, treatment decisions and outcomes depend on millimeter-level precision at every stage, from diagnostic assessment through surgical planning to intraoperative execution, as even minor deviations can result in complications such as extensor tendon damage [33]. The inability of current MLLMs to reliably characterize displacement and articular involvement suggests that these models do not yet operate at the level of anatomical precision required to meaningfully support surgical planning.

Beyond diagnostic performance, the present findings also carry methodological implications. The results suggest that reliability metrics such as Fleiss’ κ should be reported alongside sensitivity and accuracy, and that minimum reproducibility thresholds should be established before integration into diagnostic workflows. Current MLLMs behave less like deterministic diagnostic systems and more like probabilistic observers whose outputs vary across repeated evaluations of identical radiographs.

### 4.6. Limitations

Several limitations should be considered when interpreting these results.

1. The evaluation was restricted to fracture-positive cases. All 50 radiographs showed confirmed distal radius fractures, precluding assessment of specificity, false-positive rates, or negative predictive value. This constitutes a substantial constraint on clinical generalizability, as real-world screening populations include a majority of fracture-negative cases. The reported sensitivity values therefore reflect performance under constrained conditions and should not be extrapolated to mixed clinical populations. This design was chosen to enable focused evaluation of characterization subtasks (intra-articular extension, displacement), which require fracture-positive cases.
2. The sample size of 50 cases, while comparable to prior MLLM evaluation studies, limits the precision of performance estimates. Descriptively meaningful accuracy differences between models should be interpreted with caution.
3. Reference classifications were derived from the source database [26] and subsequently validated by consensus review among all six authors. However, no formal inter-rater reliability analysis of the reference standard was performed, and independent re-annotation by blinded external experts was not conducted. The diagnostic consensus may therefore carry unmeasured observer bias.
4. All models were accessed via their official web interfaces using default inference settings. Inference parameters such as temperature, top-p, and decoding strategies were neither accessible nor controlled through the web interfaces, which may limit strict reproducibility of individual outputs. The observed inter-run variability therefore reflects both inherent model stochasticity and uncontrolled inference configurations. This approach was chosen to reflect real-world user-facing deployment conditions rather than optimized laboratory settings.
5. Radiographic images were sourced from publicly available educational resources [26]. Given the widespread use of such datasets in the pre-training of contemporary foundation models, the possibility of training data contamination or partial memorization cannot be excluded. This represents a structural limitation of evaluating proprietary models with unknown training corpora. However, the substantial inter-run variability observed across most models argues against pure memorization and suggests generative processing rather than deterministic retrieval. This limitation applies broadly across all evaluated architectures and does not differentially explain the observed performance differences.
6. The evaluation employed a zero-shot prompting strategy without task-specific instructions, chain-of-thought reasoning, or few-shot examples. While this approach ensures standardized and reproducible evaluation conditions, alternative prompting strategies may yield different performance profiles. Zero-shot prompting was selected to isolate pre-trained visual reasoning capabilities and to reflect the most common user interaction pattern.
7. All prompts were presented in English. Performance may differ for prompts in other languages, and the results should not be generalized across linguistic contexts without dedicated evaluation.
8. MLLM capabilities evolve rapidly through provider-side updates that may alter model behavior without explicit disclosure. The present results reflect model versions available in March 2026 and represent a cross-sectional snapshot that may not generalize to future iterations.
9. The evaluation focused on categorical and continuous output accuracy and did not assess the quality of explanatory reasoning, clinical narrative, or confidence calibration. High accuracy on a binary classification task does not imply that the model’s underlying reasoning process is clinically sound, and future studies should incorporate qualitative analysis of model-generated rationales.
10. No standardized skeletal age assessment method such as the Greulich and Pyle atlas [16,34] was incorporated into the prompt; the zero-shot design was intended to probe unprompted feature extraction. Future evaluations could explore atlas-guided or retrieval-augmented prompting strategies, which may yield different results.

Finally, this study assessed radiographic interpretation in isolation and did not evaluate performance within integrated clinical scenarios involving patient history, physical examination findings, or treatment planning. Radiographic fracture assessment in clinical practice occurs within a broader decision-making context that the present evaluation design does not capture.

## 5. Conclusions

Contemporary MLLMs demonstrate strong but task-dependent performance on distal radius fracture radiographs. While two models achieved fracture detection sensitivity above 90%, clinically critical subtasks such as intra-articular extension and displacement assessment remain unresolved. Substantial dissociations between diagnostic performance and inter-run reliability were observed, indicating that single-run evaluations overestimate model robustness. Multi-run evaluation frameworks with explicit reporting of reliability metrics alongside sensitivity and accuracy should be considered in future studies assessing MLLM performance in medical imaging. Future validation on larger mixed cohorts including fracture-negative cases and clinically integrated scenarios will be necessary to determine whether these systems can meaningfully support musculoskeletal radiology workflows.

## Figures and Tables

**Table 1:** Sensitivity (%) across five inference runs per model for fracture detection in a fracture-positive cohort (*n* = 50 cases).

Model	Run 1	Run 2	Run 3	Run 4	Run 5	Mean (Range)	95% CI
Claude Opus 4.6	94.0	96.0	92.0	92.0	92.0	93.2 (92.0–96.0)	[89.4–95.7]
ChatGPT 5.3	72.0	72.0	76.0	64.0	76.0	72.0 (64.0–76.0)	[66.1–77.2]
Gemini 3.1 Pro	100.0	98.0	100.0	100.0	100.0	99.6 (98.0–100.0)	[97.8–99.9]
Grok 4.1	52.0	40.0	36.0	38.0	32.0	39.6 (32.0–52.0)	[33.7–45.8]
ERNIE 5.0	30.0	40.0	74.0	34.0	52.0	46.0 (30.0–74.0)	[39.9–52.2]

Values represent sensitivity per run. 95% CI = Wilson score confidence interval, calculated over pooled observations across five runs (*n* = 250 per model).

**Table 2:** Diagnostic accuracy (%) across five inference runs per model for intra-articular extension classification (*n* = 50 cases).

Model	Run 1	Run 2	Run 3	Run 4	Run 5	Mean (Range)	95% CI
Claude Opus 4.6	46.0	50.0	54.0	54.0	58.0	52.4 (46.0–58.0)	[46.2–58.5]
ChatGPT 5.3	48.0	48.0	56.0	66.0	60.0	55.6 (48.0–66.0)	[49.4–61.6]
Gemini 3.1 Pro	54.0	46.0	52.0	62.0	60.0	54.8 (46.0–62.0)	[48.6–60.9]
Grok 4.1	58.0	42.0	54.0	56.0	58.0	53.6 (42.0–58.0)	[47.4–59.7]
ERNIE 5.0	56.0	54.0	42.0	56.0	50.0	51.6 (42.0–56.0)	[45.4–57.7]

Values represent percentage accuracy per run. 95% CI = Wilson score confidence interval, calculated over pooled observations across five runs (*n* = 250 per model).

**Table 3:** Diagnostic accuracy (%) across five inference runs per model for fracture displacement classification (*n* = 50 cases).

Model	Run 1	Run 2	Run 3	Run 4	Run 5	Mean (Range)	95% CI
Claude Opus 4.6	68.0	70.0	66.0	74.0	74.0	70.4 (66.0–74.0)	[64.5–75.7]
ChatGPT 5.3	64.0	50.0	74.0	68.0	70.0	65.2 (50.0–74.0)	[59.1–70.8]
Gemini 3.1 Pro	68.0	76.0	66.0	68.0	70.0	69.6 (66.0–76.0)	[63.6–75.0]
Grok 4.1	48.0	32.0	36.0	32.0	26.0	34.8 (26.0–48.0)	[29.2–40.9]
ERNIE 5.0	44.0	48.0	70.0	46.0	52.0	52.0 (44.0–70.0)	[45.8–58.1]

Values represent percentage accuracy per run. 95% CI = Wilson score confidence interval, calculated over pooled observations across five runs (*n* = 250 per model).

**Table 4:** Inter-run reliability (Fleiss’ κ) by model and diagnostic task.

Model	Fracture	Intra-Articular	Displacement	Sex	Interpretation Range
Claude Opus 4.6	0.621	0.782	0.653	0.655	Substantial
ChatGPT 5.3	0.464	0.376	0.437	0.273	Fair–Moderate
Gemini 3.1 Pro	≈0.00 *	0.551	0.398	0.495	Slight–Moderate
Grok 4.1	0.130	0.081	0.089	0.032	Slight
ERNIE 5.0	0.122	0.150	0.154	0.044	Slight

κ values were interpreted according to Landis and Koch (1977): <0.20 slight, 0.21–0.40 fair, 0.41–0.60 moderate, 0.61–0.80 substantial, and 0.81–1.00 almost perfect agreement [27]. * Gemini 3.1 Pro achieved near-perfect fracture detection sensitivity (99.6%). Because the study cohort contained only fracture-positive cases, the resulting extreme prevalence imbalance causes expected chance agreement (P_e_) to approach 1. In such situations Fleiss’ κ may underestimate reproducibility despite high observed agreement, a phenomenon known as the “kappa paradox” (see Section 2.6).

**Table 5:** Exploratory results for demographic attribute inference across five runs per model (*n* = 250 observations per model).

Model	Age MAE (Years)	Sex Accuracy (%)	Sex 95% CI
Claude Opus 4.6	13.04 ± 10.40	54.0	[47.8–60.1]
ChatGPT 5.3	19.43 ± 15.22	57.6	[51.4–63.6]
Gemini 3.1 Pro	14.16 ± 11.32	64.4	[58.3–70.1]
Grok 4.1	19.84 ± 14.91	53.2	[47.0–59.3]
ERNIE 5.0	20.79 ± 15.31	43.6	[37.6–49.8]

MAE = Mean Absolute Error (mean ± SD). Majority-class baseline for sex prediction: 56.0% (male). 95% CI = Wilson score confidence interval, calculated over pooled observations across five runs (*n* = 250 per model).

## Data Availability

Radiographic images were obtained from publicly available educational resources (https://radiopaedia.org; (accessed on 2 March 2026)) and used exclusively for model evaluation. The sources of all images are documented in the reference list. The original image files are not redistributed due to licensing terms and third-party copyright considerations. Derived data, including aggregated performance metrics and model outputs, are available from the corresponding author upon request.

## Supplementary Materials

The following supporting information can be downloaded at: https://www.mdpi.com/article/10.3390/diagnostics16081171/s1. Supplementary Material S1: Standardized zero-shot prompt used for all models and inference runs.

## Abbreviations

The following abbreviations are used in this manuscript:

AI	Artificial intelligence
API	Application programming interface
CC BY-NC-SA	Creative Commons Attribution-NonCommercial-ShareAlike
CLAIM	Checklist for Artificial Intelligence in Medical Imaging
CNN	Convolutional neural network
GenAI	Generative artificial intelligence
JPEG	Joint Photographic Experts Group
κ	Kappa coefficient
LLM	Large language model
MAE	Mean Absolute Error
MLLM	Multimodal large language model
NPV	Negative Predictive Value
Pe	Expected chance agreement
PNG	Portable Network Graphics
SD	Standard Deviation
