# Supplementary material for: AI in Hand and Wrist Radiography: Multimodal Large Language Models for Distal Radius Fracture Detection and Characterization
Source: Diagnostics (Basel). 2026 Apr 15;16(8):1171. doi: 10.3390/diagnostics16081171 (PMC13115499; doi:10.3390/diagnostics16081171)
Supplement: Supplementary file 1 [file diagnostics-16-01171-s001.zip › diagnostics-4231160-supplementary.pdf]

Supplementary S1: Prompt Used for Model Inference

You are assisting with the structured assessment of a wrist radiograph.
Carefully analyze the provided radiograph of the distal radius and answer the following questions based solely on visible radiographic findings.

Please provide the following outputs:
1. Fracture detection
Does the radiograph show a fracture of the distal radius?
Answer: Yes or No

2. Intra-articular involvement
If a distal radius fracture is present, determine whether the fracture extends into the radiocarpal joint surface.
If no fracture is present, answer "No".
Answer: Yes or No

3. Fracture displacement
If a distal radius fracture is present, determine whether the fracture is displaced.
If no fracture is present, answer "No".
Answer: Yes or No

4. Estimated patient age
Provide your best estimate of the patient's age in years.

5. Estimated patient sex
Based on skeletal morphology visible in the radiograph, estimate the patient's sex.
Answer: Male or Female

Output Format
Fracture: Yes/No
Intra-articular: Yes/No
Displacement: Yes/No
Estimated age: [number]
Estimated sex: Male/Female

Important Instructions
- Base all responses solely on the radiographic image.
- Do not provide explanations or additional commentary.
- Provide only the requested structured output.
